# *In vitro* studies of the renin-angiotensin system in human adipose tissue/adipocytes and possible relationship to SARS-CoV-2: a scoping review

**DOI:** 10.1080/21623945.2023.2194034

**Published:** 2023-03-27

**Authors:** Ryan Ting, Heidi Dutton, Alexander Sorisky

**Affiliations:** a Faculty of Medicine, University of Ottawa, Ottawa, Canada; b Department of Medicine, University of Ottawa, Ottawa, Canada; c The Ottawa Hospital/Ottawa Hospital Research Institute, Ottawa, Canada; d Department of Biochemistry, Microbiology, and Immunology, University of Ottawa, Ottawa, Canada

**Keywords:** Renin-angiotensin system, adipose tissue, human, in vitro studies

## Abstract

The renin-angiotensin system (RAS) operates within adipose tissue. Obesity-related changes can affect adipose RAS, predisposing to hypertension, type 2 diabetes, and possibly severe COVID-19. We evaluated the *in vitro* research on human adipose RAS and identified gaps in the literature. Medline (Ovid), Embase (Ovid), Web of Science, Scopus, and 1findr were searched to identify relevant studies. Fifty primary studies met our inclusion criteria for analysis. Expression of RAS components (*n* = 14), role in differentiation (*n* = 14), association with inflammation (*n* = 15) or blood pressure (*n* = 7) were investigated. We found (1) obesity-related changes in RAS were frequently studied (30%); (2) an upswing of articles investigating adipose ACE-2 expression since the COVID-19 pandemic; (3) a paucity of papers on AT2R and Ang (1–7)/MasR which counterbalance Ang II/ART1; (4) weight loss lowered adipose ACE-2 mRNA expression; and (5) angiotensin receptor blockers (ARBs) reduced deleterious effects of angiotensin II. Overall, these studies link Ang II/ATR1 signalling to impaired adipogenesis and a pro-inflammatory dysfunctional adipose tissue, with ATR1 blockade limiting these responses. ACE-2 may mitigate Ang II effects by converting it to Ang(1–7) which binds MasR. More work is needed to understand adipose RAS in various pathologic states such as obesity and COVID-19 infection.T.

## 1. Introduction

The renin-angiotensin system (RAS) is involved in the regulation of blood pressure, electrolyte balance, inflammation, and tissue remodelling. [1] Angiotensinogen (AGT) is released from the liver and is cleaved to form angiotensin I (Ang I) by renin secreted from the kidneys. Ang I is then processed by angiotensin converting enzyme-1 (ACE-1) to form angiotensin II (Ang II). Other non-RAS enzymes such as cathepsin D can also cleave AGT to produce Ang II [2]. Ang II interacts with angiotensin type 1 receptors (AT1R) and angiotensin type 2 receptors (AT2R) to exert its physiological effects. This includes vasoconstriction of vascular smooth muscle cells and aldosterone release from the adrenal cortex, leading to sodium and water retention and an increase in blood pressure [2]. Ang II is cleaved by angiotensin converting enzyme-2 (ACE-2) to Ang (1–7) which interacts with Mas receptors (MasR) to counteract the vasoconstrictor-promoting effects of Ang II [3]. Several tissues possess their own RAS and generate angiotensin peptides that act locally or enter the circulation to exert distant effects. Adipocytes produce and secrete all of the described RAS components [4].

Adipose tissue is a multi-depot organ and plays a role in energy regulation and inflammation/immunity. RAS in adipose tissue plays an important role in adipogenesis as well as in lipid/glucose metabolism and inflammation [5]. Obesity-associated pro-inflammatory and oxidative stress results from adipocyte hypertrophy and associated cellular hypoxia [6]. This increases macrophage infiltration into adipose tissue promoting expression of inflammatory mediators and dysregulation of adipokines such Ang II. An increase in Ang II has been suggested to contribute to obesity-associated hypertension and insulin resistance [5].

Adipose RAS may potentially contribute to the body’s response to viral infections. In 2003, a global outbreak of severe respiratory syndrome caused by coronavirus SARS-CoV-1 resulted in 916 deaths [7]. From 24 January 2020 to 30 July 2021, the global pandemic of COVID-19 caused by coronavirus SARS-CoV-2 has resulted in 4,248,387 deaths [8]. The SARS-CoV-1 and SARS-CoV-2 viruses selectively bind to ACE-2 to infect human cells and may reduce ACE-2 locally or systemically [9]. Recently, SARS-CoV-2 RNA has been detected in adipocytes [10]. The reduction of ACE-2 could disrupt Ang II/AT1R and Ang (1–7)/MasR homoeostasis [11]. Increased Ang II levels have been associated with vasoconstriction, inflammation, cell proliferation, hypertrophy, fibrosis, and tissue remodelling [12]. Individuals with obesity are at high risk for severe COVID-19 infections, and it has been suggested higher levels of ACE-2 in adipose cells may lead to this tissue acting as a reservoir for viral spread within an individual [9]. Therefore, reviewing what is known about the human cellular function of adipose RAS is timely and pertinent.

We undertook a scoping review to provide a preliminary assessment of this broad and complex emerging topic. The primary objective was to provide an overview of *in vitro* research investigating the function of RAS in adipose tissue/adipocytes derived from human cell lines or human adipose tissue, and its possible association with SARS-CoV-2. Our secondary objective is to identify uncertainties or gaps in the existing literature, providing a framework for future research initiatives.

## 2. Methods

### 2.1. Methodological approach

The review process was based on the methodological framework proposed by Arksey and O’Malley [13]. The review was conducted in accordance with the Preferred Reporting Items for Systematic reviews and Meta-Analyses extension for Scoping Reviews (PRISMA-ScR) checklist guidelines [14,15]. Registration for scoping reviews on PROSPERO (the NIH international prospective register of systematic reviews) was not available at the time this article was prepared.

### 2.2. Research question and key concepts

What are the extent and nature of *in vitro* research investigating the renin-angiotensin system in human adipose tissue/adipocytes? What is the possible association of human adipose RAS with SARS-CoV-2?

### 2.3. Database search

To identify relevant documents, the following bibliographic databases were searched from inception to 24 June 2020: MEDLINE, EMBASE, PubMed, Web of Science, Scopus, and 1findr. The search strategies were drafted in consultation with an information specialist (KF) and further refined through team discussion with all authors. A pretested combination of keywords and MESH terms were used based upon the identified core concepts of the research question: (1) adipose tissue/cells; (2) renin-angiotensin system; (3) *in vitro*. A detailed search strategy for MEDLINE (Ovid) is shown in Supporting Information S1. Additional sources of information included reference lists from retrieved papers [16].

We also searched PubMed on 30 July 2021 using the terms ‘human’, ‘adipose’, and ‘ACE-2’ to identify the ongoing activity of ACE-2 publications after the first search was completed.

### 2.4. Study selection

Eligibility criteria were established based on research questions and pretested in PubMed. Primary research articles written in English that fulfiled the following criteria were included in the analysis: (i) a component of the renin-angiotensin system was investigated; (ii) at least one indicator of cellular expression of RAS in adipose tissue/adipocytes was identified; (iii) studies were performed with human adipose/adipocytes or human cell lines *in vitro*; (4) studies were related to adipose tissue or adipocyte function. We did not include animal cell studies in this defined scoping review, as they are not as relevant and immediate as human cell studies are to the understanding of clinical disease states.

Studies excluded from this study were as follows: (i) manual duplicates (ii) review articles and meta-analysis; (iii) studies for which the full-text article did not exist such as a conference/abstract; (iv) editorials/commentaries; (v) *in vivo* human studies that did not include isolated adipose tissue/adipocytes or human adipose cell lines; (vi) studies performed with other human cells that did not include adipose tissue/adipocytes; (vii) *in vitro* studies which did not examine the effect of RAS in human adipose tissue/adipocytes; (viii) *in vitro* studies involving adipose tissue/adipocytes from animal-derived tissue (transgenic animals), cells, and cell lines; (ix) studies reported in languages for which no English language translation was available.

### 2.5. Screening

All the retrieved records from the databases and hand-searches were imported in Zotero reference management database (version 5.0 Corporation for Digital Scholarship, Roy Rosenzweig Center for History and New Media, George Mason University, Fairfax, VA, USA). The records were then converted into a RIS format for import into Covidence systematic review software, Veritas Health Innovation, Melbourne, Australia (available at www.covidence.org) which was used for screening. In order to minimize bias, titles and abstracts of studies identified by the systematic search were screened for relevance by two independent investigators, AS and RT. Relevant articles identified through the screen were reviewed for complete assessment of eligibility criteria. Discrepancies were resolved through discussion and consensus. A third reviewer, HD, was available to resolve any conflicts.

### 2.6. Data extraction

Prior to the search, a data extraction form was designed to facilitate interpretation, comparison, and synthesis of the findings from the included studies. Pretesting of the data extraction form was performed by RT on five papers, which then led to further refinement of the form after discussion with the other authors. RT conducted the extraction. AS verified the data extraction to ensure accuracy and reproducibility. The final version of the form included: authors, publication year, paper title, journal, country of senior/corresponding author, purpose, study population, cell type(s) isolated/source of tissues, components of RAS studied, methodology (study design), experimental treatments, key findings, and sponsorship.

## 3. Results

### 3.1. Study selection

**Figure 1.**
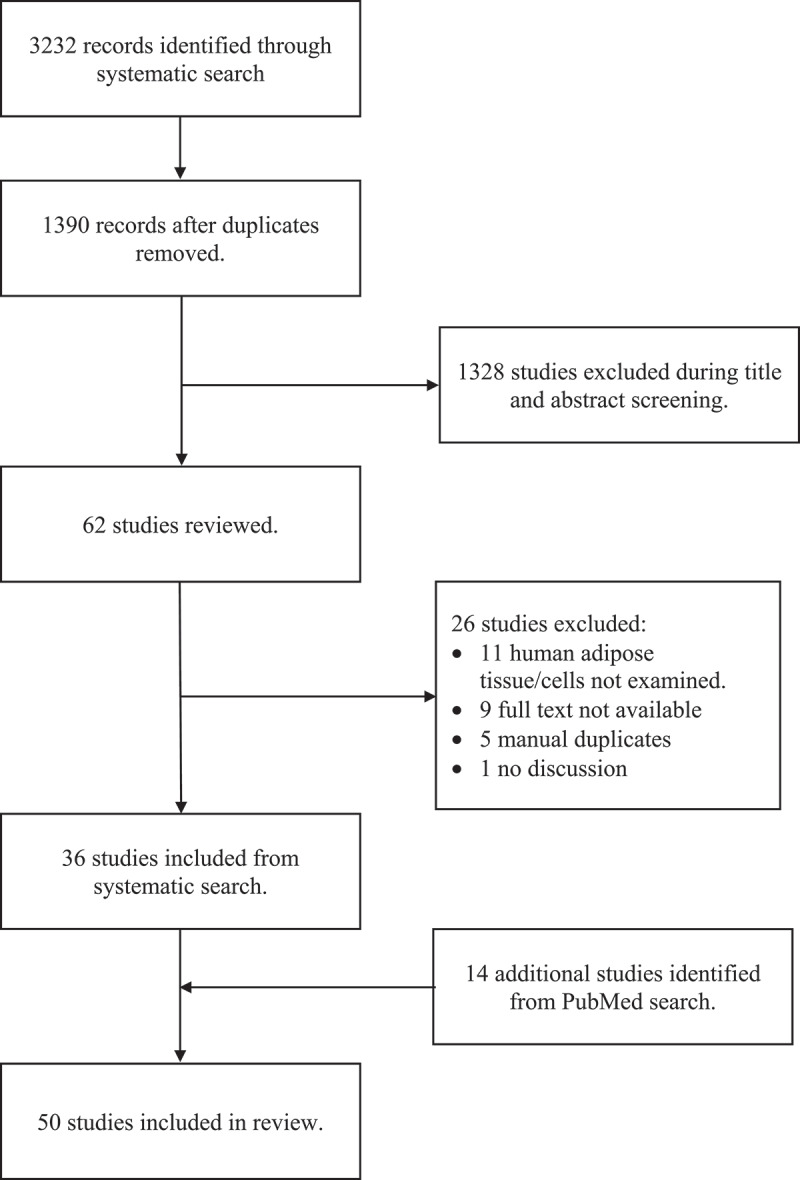
Preferred Reporting Items for Systematic Reviews and Meta-Analysis (PRISMA) flow diagram.

Our initial search strategy conducted on 24 June 2020 identified 3232 articles. Removal of duplicates by Covidence resulted in 1390 unique articles. After screening titles and abstracts for relevance, 62 studies were eligible for full-text review. Of these articles, 26 were excluded for the following reasons: not conducted in human adipose tissue/cells (*n* = 11), full text not available (*n* = 9), manual duplicates (*n* = 5), no discussion section (*n* = 1). This left 36 studies to review. A PubMed search was conducted on 30 July 2021 to capture articles for ACE-2 published after our initial database search. Additional 14 studies were retrieved. Therefore, 50 studies were included in this scoping review [17–66] (Figure 1).

### 3.2. Study characteristics

The publication period of the 50 eligible studies ranged from 2001 to 2021. Of these, 14 studies investigated expression of RAS components, 14 studies investigated RAS and differentiation, 15 studies investigated RAS and inflammation, and 7 studies investigated RAS and blood pressure. Tables 1–4 contain specific details of each study such as those related to age, gender, depot of adipose tissue and country.

**Table 1.** Expression of RAS components in human adipose tissue *in vitro* studies (*N* = 14).

Reference	Study population & Country of Corresponding Author	Cell type(s) isolated/Source of tissues	RAS Component(s) studied	Key Findings
Archard et al. 2007 [30]	Females 18–55 yrs.: BMI 21.0 ± 2.2 (*n* = 14); BMI 37.9 ± 5.2 (*n* = 17) (France)	Stromal preadipocytes from biopsies of abdominal SAT and omental VAT.	AGT, and Renin receptor (RenR)	RenR was localized in the cell periphery. Renin binding to RenR of preadipocyte cells *in vitro* stimulated intracellular-signalling pathways and potentiated AGT to Ang I generation. RenR expression was higher in VAT.
Couselo-Seijas et al. 2021 [24]	36 patients who underwent open-heart surgery. Age 70 ± 0.8 yrs. Females (*n* = 11); Male (*n* = 25); Obese and non-obese BMI 30 ± 0.95; diabetic and non-diabetic patients. (Spain)	Epicardial fat (EAT) and SAT biopsies; Stromal vascular cells (*n* = 8).	ACE-1, and ACE-2	Similar levels of ACE-1 in EAT and SAT. EAT expressed higher levels of ACE-2 and lower ADAM17 mRNA than SAT. EAT and SAT from cardiac patients with obesity and T2DM had the highest levels of ACE-2 mRNA. Diabetic patients treated with ARBs had higher ACE-2 mRNA levels than diabetic patients not treated with ARBs.
de Ligt et al. 2021 [26]	Impaired glucose metabolism. BMI 31 ± 0.8 (*n* = 36) (Netherlands)	Abdominal SAT biopsy.	AGT, AT1R, ACE-1, and ACE-2	Expression of AGT, ACE-1, ACE-2 and AT1R mRNA in group treated for 26 weeks with valsartan was not significantly different from the placebo group.
Desterke et al. 2021 [25]	Transcriptome datasets from Gene Expression Omnibus (GEO) website. (France)	Human mesenchymal stem cells (hMSC).	ACE-2	ACE-2 mRNA was highly expressed in adult bone marrow, adipose tissue, and umbilical cord-derived hMSC.
Fain et al. 2007 [28]	Female avg. 39 yrs. Obese: Mean BMI 32.9 (*n* = 10) Morbidly obese: Mean BMI 46 (*n* = 12). (USA)	Explants of abdominal SAT, and omental VAT. (Non-fat cell and adipocyte fractions)	ACE-1	The majority of the *in vitro* release of ACE-1 was from non-fat cells. Higher levels of ACE-1 are released by adipose tissue from subjects with morbid obesity. Higher levels of ACE-1 released by omental VAT versus SAT explants.
Fain et al. 2008 [29]	Female avg. 40 yrs. Morbidly obese avg. BMI 46 (USA)	Stromovascular portion of omental VAT.	AGT, AT1R, ACE-1 and RenR	Adipocyte versus non-adipocyte mRNA: AGT is higher in adipocytes and RenR lower in adipocytes. Adipocyte vs. preadipocyte mRNA: AGT & AT1R higher in adipocytes; RenR same; ACE-1 lower in adipocytes.
Favre et al. 2021 [23]	Non-obese patients with and without COVID-19. (France)	VAT and SAT	ACE-2	Positive correlation between ACE2 mRNA and BMI in VAT, but not SAT. Higher ACE2 with COVID-19 ACE-2 was not related to age and sex.
Han et al. 2020 [17]	RNAseq transcriptome and sex data from the genotype-tissue expression (GTEx) and the Cancer Genome Atlas (TCGA). (China)	Human tissues	ACE-2	ACE-2 mRNA levels were highest in the small intestines. ACE-2 mRNA levels were high in salivary glands, testicular, kidney, heart, thyroid and adipose tissue. No significant sexual differences in ACE2 gene expression.
Kerslake et al. 2020 [18]	Genotype-Tissue Expression (GTEx), the Cancer Genome Atlas (TCGA), and GEPIA (http://gepia.cancer). (United Kingdom)	Human tissue	ACE-2	ACE-2 mRNA was expressed in adipose tissue.
Kristem et al. 2021 [21]	Gene Expression Omnibus (GEO) Data Sets. Females avg. 47 years: Obese (*n* = 16); non-obese (*n* = 16) (USA)	SAT pre- and post- Roux-en-Y gastric bypass (RYGB)	ACE-2	After RYGB, mRNA expression of ACE-2 in SAT decreased. ACE-2 in SAT was lower in post-RYGB patients vs non-obese matched controls.
Li, L et al. 2020 [22]	Age>18 yrs. Obese (*n* = 156); Female (*n* = 120); Male (*n* = 36) (Germany)	SAT biopsy	ACE-2	No difference in ACE-2 mRNA expression for males and females and differences in age. ACE-2 mRNA expression was reduced in insulin-resistant subjects. ACE-2 mRNA expression was correlated with adipose leptin production. ACE-2 mRNA expression was lower after BMI reduction and improved insulin sensitivity.
Li, M et al. 2020 [19]	GTEx datasets (RSEM normalized) from UCSC Xena project, TCGA datasets from Genomic Data Commons Data Portal. (China)	Human tissues	ACE-2	ACE-2 mRNA expression levels in adipose tissue were high in comparison to other tissue types. No difference in ACE2 mRNA expression between males and females or between younger and older persons in any tissues.
Liu, et al. 2021 [20]	GTEx database; COVID-19 host genetics initiative 2020. (Sweden)	Human tissues	ACE-2	ACE-2 mRNA was found in adipose tissue.
Mackay et al. 2006 [27]	Donors aged 22–27 yrs. hMSC and medullary adipocytes. Pooled SAT preadipocytes from female donors (*n* = 6), BMI 24.5-28.2 (Zen-Bio, Inc.) (USA)	Medullary adipocytes and hMSC derived adipocytes from donor iliac crest. Commercial SAT preadipocytes.	AGT	Medullary, SAT and hMSC derived adipocytes showed no differences in expression for AGT mRNA.

**Table 2.** RAS components and differentiation of human adipose tissue-derived cells *in vitro* studies (*N* = 14).

Reference	Study population & Country of Corresponding Author	Cell type(s) isolated/Source of tissues	RAS Component(s) studied	Key Findings
Brücher et al. 2007 [35]	Females (*n* = 13), Males (*n* = 11); BMI<30 (*n* = 15), BMI≥30 (*n* = 9). (Chile)	Stroma-vascular preadipocytes from greater omental VAT.	Ang II, AT1R, and AT2R	Ang II reduced recruitment of undifferentiated cells to undergo adipogenic differentiation *in vitro*. Inhibition of adipogenic differentiation by Ang II correlated directly with BMI. The effect of Ang II was greater on preadipocytes from obese donors. Inhibition of adipogenesis by Ang II was reversed by losartan (AT1R inhibitor). CGP42112A (AT2R inhibitor) did not reverse Ang II inhibition of adipogenesis.
Dünner et al. 2013 [37]	Female donors (*n* = 14); BMI 34 ± 5.7; Simpson-Golabi-Behmel syndrome (SGBS) neonates. (Chile)	Preadipocytes from omental VAT and SGBS neonates.	Ang II	Ang II signalling in human preadipocytes involved an ERK1/2-dependent attenuation of Akt activity.
Engeli et al. 2004 [34]	Healthy females 25–60 yrs.; BMI 22–35. (Germany)	Mammary SAT adipocytes and preadipocytes	Ang II	Insulin and Ang II increased NO production by human preadipocytes *in vitro*.
Fuentes et al. 2010 [38]	Non-obese subjects. (Chile)	Preadipocytes from human omental fat.	Ang II	Ang II reduced adipogenesis during the first 48 h of differentiation induction. Ang II increased phosphorylated PPARϒ and ERK1/2 after induction of adipogenesis. Inhibition of MEK1 activity prevented ERK1/2 phosphorylation and blocked the anti-adipogenic effect of Ang II.
Gaafar et al. 2015 [44]	Patients undergoing anterior abdominal wall surgeries. (Egypt)	ad-MSCs from abdominal wall biopsy.	Ang II	Ang II and 5-azacytidine were unsuccessful in inducing trans-differentiation of human ad-MSCs into cardiomyocytes *in vitro*.
Janke et al. 2002 [36]	Healthy females 20–60 yrs.; BMI 22–35. (Germany)	Preadipocytes and mature adipocytes from mammary SAT.	AGT, Ang II, AT1R, AT2R, and ACE-1	Adipogenesis was associated with an increase in mRNA expression of AGT, renin, ACE-1, and AT1. Incubation with Ang II or AGT resulted in a dose-dependent inhibition of adipogenesis. AT1R blockage with irbesartan increased lipid accumulation (increased cells and increased intracytoplasmic lipid accumulation). Co-culture of preadipocytes with adipocytes inhibited adipogenesis. Effects were abolished by AT1R blocker.
Janke et al. 2006 [39]	Healthy females 20–60 yrs.; BMI 22–35; Healthy males; BMI>30. (Germany)	Preadipocytes and mature adipocytes from mammary SAT.	AT1R	AT1R blockers (telmisartan, irbesartan and losartan) induced adipogenesis and PPAR-ϒ activation which increased mRNA and protein expression of lipoprotein lipase and adiponectin in preadipocytes. PPAR-ϒ antagonist (GW9662) lowered mRNA and protein expression of lipoprotein lipase and adiponectin.
Sarzani et al. 2008 [42]	Males (*n* = 8); Post-menopausal females (*n* = 7) Perirenal avg. age 67.2 yrs (*n* = 16). Omental avg. age 69.3 yrs (*n* = 9). (Italy)	Perirenal adipocytes and preadipocytes. Omental adipocytes and preadipocytes.	Ang II and AT1R	Omental and perirenal preadipocyte proliferation was stimulated by Ang II and inhibited by atrial natriuretic peptide (ANP). Valsartan (ARB) blocked this effect of Ang II. AT1R, but not AT2R mRNA was expressed in omental adipose tissue.
Schling et al. 2004 [31]	Healthy females and males aged 19–63 yrs. (Germany)	Human SAT preadipocytes from abdominal or breast surgery.	Ang II, AT1R, and AT2R	AT1R and AT2R were present in human preadipocytes before, during and after differentiation into adipocytes. AT1R mRNA declined sharply during adipogenesis; protein levels remain unchanged. AT2R mRNA and protein levels increased during adipogenesis.
Song et al. 2013 [43]	Adult female (China)	ADSCs from abdominal SAT	Ang II	Ang II and 5-azacytidine were able to induce trans-differentiation of 20% of the human ad-MSCs into cardiomyocytes-like cells *in vitro*.
Sysoeva et al. 2017 [32]	Donors avg. BMI 23.5 ± 2.4 (*n* = 18) (Russia)	ADSCs from abdominal SAT	Ang II, AT1R, AT2R, and ACE-1	Human SAT contained a subpopulation of ADSCs constantly co-expressing AT1R and AT2R. Autocrine signalling by Ang II via AT2R increased the ability for ADSCs to undergo adipogenesis.
Than et al. 2013 [41]	Non-diabetic male subjects; BMI 25–29.9 (Zen-Bio Inc.) (Singapore)	Human SAT preadipocytes.	Ang II, AT1R, AT2R, MasR and Ang (1–7)	AT1R and MasR protein were co-expressed in human preadipocytes and adipocytes. Ang (1-7)/MasR promoted adipogenesis and increased protein expression of PPARϒ. Ang (1–7)/MasR antagonized the anti-adipogenic effect of AngII/AT1R in human preadipocytes.
Than et al. 2017 [33]	Non-diabetic subjects; BMI 25–29.9 (Zen-Bio In.) (Singapore)	Human SAT preadipocytes	Ang II, AT1R, and AT2R	The presence of AT1R and AT2R proteins was confirmed in human white adipocytes. AT2R activation by Ang II or AT2R agonists (C21) induced white adipocyte browning by increasing PPARϒ protein expression. AngII-AT2R enhanced brown adipogenesis. Increased UCP1 protein expression and O_2_ consumption was evidence of AT2R induced browning effect.
Ye et al. 2009 [40]	Commercially available cells from ScienCell Research Lab. (San Diego, CA). (China)	Human VAT preadipocytes	AGT, Ang II, AT1R, AT2R, and ACE-1.	Ang II was generated by RAS or non-RAS (cathepsin D). AGT mRNA increased during adipogenesis. Renin mRNA and ACE-1 mRNA decreased during adipogenesis. Cathepsin D mRNA increased during adipogenesis. AT1R and AT2R mRNA increased in early adipogenesis and then decreased by late adipogenesis.

**Table 3.** RAS components and inflammation *in vitro* studies (*N* = 15).

Reference	Study population & Country of Corresponding Author	Cell type(s) isolated/Source of tissues	RAS Component(s) studied	Key Findings
Blumensatt et al. 2017 [54]	Epicardial adipose tissue (EAT) biopsies from patients with and without type 2 diabetes undergoing heart surgery. (Germany)	Human EAT; Lewis rats’ cardiomyocytes	Ang II and AT1R, ACE-2	Cardiomyocytes exposed to EAT adipocyte conditioned medium (CM) from patients with type 2 diabetes reduced sarcomere shortening and increased miR-208a expression. CM from EAT adipocytes pretreated with Losartan from patients with type 2 diabetes no longer had this effect. No difference in ACE-2 between patients.
Boccara et al. 2010 [53]	Healthy participants; BMI<25. (Zen-Bio, Research Triangle Park, NC, USA). (France)	Preadipocytes from human SAT.	AGT, Ang II, and AT1R	HIV protease inhibitors (PI) increased expression of AT1R protein, AGT mRNA and amplified the effect of Ang II on ERK1/2 activity. ARBs (irbesartan, telmisartan) inhibited PI effects. Rosiglitazone normalized AT1R protein expression. GW9662 (PPAR-γ antagonist) increased AT1R protein expression and increased PI toxicity.
Pinheiro et al. 2017 [59]	Eutrophic patients, obese patients, and malnourished patients. (Brazil)	Visceral white adipose tissue (VAT)	AGT, ACE-1, and ACE-2	AGT and ACE mRNA levels were significantly higher in VAT from either obese or malnourished vs. eutrophic groups. No significant difference in ACE-2 mRNA expression between the groups. IL-6 and TNF-α mRNA expressions were significantly higher in VAT from obese or malnourishe vs. eutrophic groups.
de Oliveira et al. 2020 [57]	Commercially available cells from Sigma-Aldrich (St. Louis, MO, USA). (Brazil)	Human SAT preadipocytes.	ACE-2	Irisin regulated the expression of TLR3 genes that play a role in the regulation of ACE-2 cleavage.
Goossens et al. 2011 [45]	Male: Lean<25 (*n* = 9), normal glucose tolerance; Obese>30 (*n* = 10) impaired glucose tolerance. (Netherlands)	Abdominal SAT biopsy	Ang II	Local administration of Ang II to abdominal SAT decreased adipose tissue blood flow and PO_2_ and increased mRNA expression of inflammatory markers such as TNF-α.
Harte et al. 2005 [46]	White, nondiabetic female subjects; Age 42.3 ± 16 yrs.; BMI 29.8 ± 5.4. (United Kingdom)	Adipocytes from abdominal SAT	AGT and Ang II	Human SAT was a significant source of Ang II. Insulin increased TNF-α secretion which in turn increased the Ang II. Rosiglitazone downregulated RAS in SAT.
Li, Yi et al. 2015 [52]	Patients with active Crohn’s disease (*n* = 6): activity index>150 and CRP>10. (China)	Mesenteric adipose tissue (MAT) specimen from intestinal wall adjacent to disease involved intestine.	AT1R	MAT treated with telmisartan significantly increased mRNA expression of adiponectin and leptin and decreased mRNA expression of IL-6 and IL-17.
Menikdiwela et al. 2019 [47]	Commercially available cells purchased from LONZA (Allendale, NJ, USA). (USA)	Human mesenchymal stem cells (HMSC)	AGT, AT1R, AT2R, ACE-1	Ang II treatment increased inflammation and endoplasmic reticulum (ER) stress in adipocytes mainly via AT1R. Telmisartan reduced ER stress and inflammation. Captopril did not reduce ER stress markers.
Patel et al. 2016 [58]	Non-obese subjects, non-failing hearts vs. Obese subjects (BMI>30) with HFPEF, hypertension or transplant vasculopathy. (USA)	Epicardial adipose tissue (EAT)	ACE-2	HFPEF subjects with obesity had increased ACE-2 protein levels in EAT. HFPEF subjects with obesity had increased inflammatory markers in EAT.
Polonis et al. 2020 [49]	OSA and non-OSA subjects (*n* = 50) Commercially available primary human white preadipocytes from abdominal tissue of healthy individuals (ZenBio Inc. NC). (USA)	Abdominal SAT from subjects. Primary human white abdominal preadipocytes.	AT1R, ACE-1	Exposure of preadipocytes to Ang II increased mitochondrial ROS production, DNA damage, and a higher percentage of SA-β-gal positive cells (senescence) in preadipocytes. Treatment of preadipocytes with ARB (losartan) or ACE-1 inhibitor (captopril) inhibited the effects of Ang II.
Rasha et al. 2020a [55]	Human breast cancer cells from ATCC (Manassas, VA, USA). Bone-marrow-HMSC from Lonza (Allendale, NJ, USA). (USA)	MDA-MB-231 TNBC and ER/PR positive MCF-7. HMSC	ACE-1	Treatment of breast cancer (BC) cells with conditioned medium (CM) from adipocytes pretreated with captopril significantly reduced expression of proinflammatory cytokines and decreased BC cell migration compared to the control group. Treatment of BC cells with CM from adipocytes pretreated with captopril and Eicosapentaenoic acid (EPA) further enhanced the positive effects of captopril.
Rasha et al. 2020b [56]	Human breast cancer cells from ATCC (Manassas, VA, USA). Bone-marrow-HMSC from Lonza (Allendale, NJ, USA). (USA)	MDA-MB-231 TNBC and ER/PR positive MCF-7. HMSC	Ang II, AT1R and ACE-1	Treatment of BC cell lines with conditioned medium (CM) from adipocytes pretreated with Ang II significantly increased inflammatory tumour promoters in BC cells. Treatment of BC cell lines with conditioned medium from adipocytes pretreated with telmisartan or captopril significantly reduced markers of inflammation, fatty acid synthesis and angiogenesis in BC cell lines.
Skurk et al. 2001 [50]	Females 18–45 yrs.; BMI<26. (Germany)	Mammary SAT Preadipocytes	Ang II and AT1R	Exposure of human adipocytes to Ang II resulted in a dose- and time-dependent stimulation of PAI-1 release. Candesartan inhibited action of Ang II indicating effect was mediated by AT1R.
Skurk et al. 2004 [48]	Females 18–50 yrs.; BMI≤26. (Germany)	Mammary SAT Pre-adipocytes	Ang II, AT1R and AT2R	Ang II significantly increased IL-6 and IL-8 production and release by a NF-_k_B-dependent pathway. Pro-inflammatory action of Ang II was primarily mediated by AT1R versus AT2R.
Skurk et al. 2005 [51]	Healthy young or middle-aged women; BMI 20–27 (Germany)	Stromal cells isolated from mammary SAT	Ang II and AT1R	Ang II increased leptin secretion into the culture medium in a dose- and time-dependent fashion.

**Table 4.** RAS components and blood pressure *in vitro* studies (*N* = 7).

Reference	Study population & Country of Corresponding Author	Cell type(s) isolated/Source of tissues	RAS Component(s) studied	Key Findings
Ehrhart-Bornstein et al. 2003 [66]	Healthy females 20–35 yrs. (*n* = 10); BMI 21.4–29.2 (Germany)	Mammary SAT adipocytes. Adrenal NCI-H295R cells	Ang II and AT1R	Stimulation of aldosterone secretion by NCI-H295R cells was not mediated by Ang II.
Gorzelniak et al. 2002 [64]	Hypertensive females BP > 130/80 (*n* = 30): Obese (BMI > 30); Lean (BMI < 25); abdominal biopsy. Healthy females (*n* = 4) 34–60 yrs.; BMI 23–31; breast reduction. (Germany)	Adipocytes from: SAT needle biopsy of periumbilical region. Mammary SAT.	AGT, Ang II, AT1R, AT2R, renin and ACE-1	Detected AGT, renin, ACE-1, AT1R and AT2R mRNA expression in adipocytes but AT2R was barely detectable. Insulin, thyroxine, oestradiol, and Ang II had no significant effect on the expression of RAS genes studied. Hydrocortisone increased AT1R mRNA expression. AGT mRNA expression was significantly lower in obese vs. non-obese. Renin, ACE-1 and AT1R mRNA expression was significantly higher in obesity vs. non-obese. Hypertensive obese subjects had the highest AT1R mRNA.
Malinowski et al. 2008 [65]	Patients undergoing surgery for stable isolated coronary artery disease. (USA)	Perivascular adipose tissue (PVT) of internal thoracic artery (ITA); Pleural adipose tissue	Ang II	PVT significantly decreased the ITA contractility response to serotonin and Ang II. PVT of ITA released nitric oxide (NO) and prostacyclin-independent anticontractile factor. Pleural adipose tissue presence did not change the ITA contractility response to serotonin and Ang II.
Park et al. 2013 [63]	Males (*n* = 21); Females (*n* = 24). (USA)	SAT and omental VAT.	AGT	Significantly higher AGT mRNA expression for −20C allele versus −20A allele in SAT. No significant difference in −20C and −20A allele AGT mRNA expression was detected in omental VAT.
Prat-Larquemin et al. 2004 [61]	Females 22–61 yrs. (*n* = 61); BMI≥28. (France)	Mature adipocytes from peri-umbilical SAT needle aspiration	AGT	Higher levels of AGT secretion in human adipocytes, with greater interindividual variation, in comparison to rat adipocytes. AGT secretion was not related to adipocyte size, BMI, blood pressure or M235T AGT gene polymorphism. Adipocyte size differed among AGT genotypes.
Sarzani et al. 2010 [62]	Consecutive patients undergoing radical nephrectomy (*n* = 35). Mean: 64.6-yr-old; BMI 27.3 (Italy)	Visceral adipose tissue (VAT).	AGT	AGT promotor variants −175 and −163 expressed higher levels of AGT mRNA in perirenal VAT than tissue from kidney cortex or kidney medulla.
Serazin et al. 2004 [60]	Males (57 ± 8.5 yrs.); BMI 26.2 ± 3.2 (*n* = 4). (France)	SAT fragments	AGT	Increased adipocyte AGT expression and secretion by cAMP suggested the sympathetic nervous system may have a role in the activation of the local RAS. cAMP increased the expression and secretion of human AGT in SAT.

#### 3.2.1. Expression of RAS components

Assessing ACE-2 expression in adipose tissue has grown in interest recently, given its possible role as a SAR-CoV-2 viral receptor with respect to obesity and susceptibility to COVID-19 infection. A variety of adipose depots were examined. Four articles detected high ACE-2 gene expression in adipose tissue relative to other tissue types [17–20] (Table 1). The other four articles examined ACE-2 mRNA expression in relation to BMI [21–24]. Kristem et al. used GEO datasets to measure ACE-2 gene expression in subcutaneous white adipose tissue (SAT) from patients with severe obesity, before and after Roux-en-Y gastric bypass (RYGB); results showed RYGB was associated with lower ACE-2 mRNA expression [21]. Similarly, Li, L et al. observed that weight loss was associated with a decline in SAT ACE-2 mRNA [22]. Favre et al. noted that expression of ACE-2 mRNA paralleled BMI in visceral adipose tissue (VAT) from overweight patients, and has been the only publication to correlate that in patients with or without COVID-19 infection [23]. Couselo-Seijas et al. found ACE-2 and ADAM17 (its cleavage enzyme) mRNA expression levels were higher in epicardial adipose tissue (EAT) from patients with type 2 diabetes mellitus and ACE-2 was highest in patients with obesity and diabetes [24]. Desterke et al. found ACE-2 mRNA was highly expressed in adipose-derived mesenchymal stem cells (MSC) [25]. deLigt et al. studies participants who received either valsartan or placebo for 26 weeks [26]. Abdominal SAT biopsies were collected before and after 26 weeks of treatment. There were no significant differences in ACE-2, AGT, ACE-1 and AT1R mRNA expression in SAT between the valsartan or placebo groups.

Four of the 14 articles examined the expression of RAS components other than ACE-2 [27–30]. Mackay et al. demonstrated that AGT mRNA expression was similar in medullary adipocytes, human mesenchymal stem cell-derived adipocytes and subcutaneous adipocytes [27]. Fain et al. observed that most of the *in vitro* release of ACE-1 from adipose tissue was by stromal non-fat cells and more ACE-1 was released by omental adipose tissue (OAT) from patients with class III obesity > 40 kg/m^2^ (mean BMI 46 kg/m^2^) versus patients with class I obesity BMI 30–35 kg/m^2^ (mean BMI 32.9 kg/m^2^) [28]. Fain et al. compared mRNA expression from adipocytes versus preadipocytes for AGT, renin receptor, AT1R and ACE-1 in OAT [29]. The expression of AGT mRNA and AT1R mRNA was greater in adipocytes than preadipocytes, whereas renin receptor mRNA expression was equal between adipocytes and preadipocytes, and the expression of ACE-1 mRNA was lower in adipocytes than preadipocytes. Archard et al. reported (pro)renin receptors were synthesized in the stromal portion of human adipose tissue (SAT and OAT) in preadipocytes and non-preadipocyte cells [30]. Renin binding to preadipocyte (pro)renin receptors increased the catalytic efficiency of AGT conversion to Ang I.

#### 3.2.2. Differentiation and RAS components

The role of RAS components has been a topic of interest. Three articles examined AT1R and AT2R expression and function using SAT human preadipocytes [31–33] (Table 2). Schling et al. noted AT2R mRNA and protein expression increased, whereas AT1R mRNA expression decreased, and protein expression of AT1R remained unchanged, during adipogenesis [31]. Sysoeva et al. [32] reported human SAT contained a subpopulation of adipose-derived mesenchymal stem cells that express AT1R and AT2R mRNA and protein, and concluded that adipogenesis required expression of both AT1R and AT2R, based on an inhibitor strategy [32]. Than et al. reported AT1R and ATR2 proteins were present in preadipocytes [33]. Ang II, acting through ATR2, induced white adipocyte browning by increasing PPAR-γ expression. Thyroid hormone T3 stimulated the protein expression of AT2R but not AT1R to further promote adipocyte browning.

Several articles examined the effect of Ang II on adipose differentiation [34–42]. Engeli et al. reported a common action of insulin and Ang II to increase nitric oxide (NO) production during differentiation of SAT preadipocytes [34]. Brücher et al. [35] demonstrated Ang II inhibited the adipogenesis of human adipocyte progenitor cells from OAT that were induced to undergo adipogenesis *in* vitro [35]. The inhibitory effect of Ang II was greater in adipocytes from obese versus non-obese donors and was blocked by ARB (losartan). Janke et al. demonstrated AGT, or Ang II, resulted in a dose-dependent inhibition of adipogenesis of preadipocytes derived from SAT [36]. The mRNA expression of AGT, renin, ACE-1, and AT1R increased during adipogenesis. Inhibition of adipogenesis by Ang II was blocked by ARB (irbesartan). Dünner et al. reported Ang II inhibited differentiation of preadipocytes from SAT and OAT into mature adipocytes [37]. Ang II exerted a greater anti-adipogenic effect on preadipocytes isolated from individuals with obesity. The anti-adipogenic effect of Ang II was associated with increased phosphorylation of ERK1/2 (a negative regulator of insulin-stimulated Akt phosphorylation) which resulted in decreased phosphorylated Akt. Fuentes et al. demonstrated Ang II reduced the adipogenesis of preadipocytes from OAT [38]. This was associated with an increase in phosphorylated ERK1/2 and an increase in phosphorylation of a key adipogenic transcription factor PPAR-γ. Janke et al. [39] found ARBs (irbesartan, losartan, and telmisartan) induced adipogenesis and activated PPAR-γ target genes in SAT [39]. Ye et al. [40] observed that, during adipogenesis of VAT-derived preadipocytes, mRNA expression of AGT and cathepsin (a non-RAS enzyme that can increase Ang II production) increased, whereas renin and ACE-1 decreased [40]. In early adipogenesis, AT1R and AT2R mRNA expression initially increased, and then decreased in the later stages. Than et al. showed that AT1R and MasR proteins were expressed in human SAT preadipocytes and adipocytes [41]. Ang (1–7)/MasR promoted adipogenesis by inhibiting the phosphorylation of PPAR-γ, which antagonized the anti-adipogenic effect of Ang II/AT1R in preadipocytes. In contrast to the other 8 articles describing the anti-adipogenic effect of Ang II, Sarzani et al. [42] found that Ang II stimulated adipogenesis. This is addressed in the Discussion.

Finally, there were 2 articles in which Ang II and 5-azacytidine (5-AZA) were used in an attempt to trans-differentiate human adipose-derived MSCs (ad-MSCs) into cardiomyocyte-like cells [43,44]. Song et al. [43] obtained a 20% trans-differentiation rate. Gaafar et al. [44] were unable to induce trans-differentiation of the ad-MSCs into cardiomyocytes using Ang II and 5-AZA.

#### 3.2.3. RAS and adipose inflammation

The focus of the investigation was the connection between RAS and adipose tissue inflammation [45–59] (Table 3). Several articles have focused on the role of Ang II in this process [45–53]. Goossens et al. administered Ang II locally into abdominal SAT which led to vasoconstriction and reduced blood flow to cause adipose tissue hypoxia [45]. This increased mRNA expression of inflammatory markers such as TNF-α. Harte et al. investigated the effects of TNF-*α*, insulin or insulin in combination with rosiglitazone on isolated adipocytes from SAT [46]. TNF-*α* increased AGT and Ang II secretion and insulin increased TNF-, AGT and Ang II secretion. Rosiglitazone, a potent PPAR-γ agonist, reduced the insulin-mediated rise in TNF-*α*, AGT and Ang II secretion. Menikdiwela et al. found Ang II increased ER stress and increased mRNA levels of NF-κB and its downstream target IL-6 in adipocytes differentiated from mesenchymal stem cells [47]. Expression of NF-κB and IL-6 mRNA was significantly reduced by ARB (telmisartan). Skurk et al. used SAT adipocytes and noted Ang II enhanced IL-6 and IL-8 protein production and release by a NF-κB-dependent pathway [48]. The proinflammatory response generated by Ang II was reduced by treatment with an ARB (candesartan). Polonis et al. demonstrated intermittent hypoxia-induced inflammation and a senescence-like phenotype (SA-*β*-gal positive cells) in SAT predipocytes. and this was accentuated by the exposure of preadipocytes to Ang II [49]. It also increased mitochondrial ROS, and treatment with an ARB (losartan) or ACE inhibitor (captopril) reduced mitochondrial ROS and the percentage of SA-*β*-gal positive cells. Skurk et al. exposed human SAT adipocytes to Ang II and found a dose- and time-dependent stimulation of the release of PAI-1, which was blocked by ARB (candesartan) [50]. In another study, Skurk et al. [51] treated SAT preadipocytes with Ang II and observed more leptin secretion in a dose- and time-dependent manner by an ERK1/2-dependent pathway, and this was inhibited by ARB (candesartan) [51]. Li et al. found that AT1R blocker and partial PPAR-γ agonist (telmisartan) promoted the mRNA expression of adiponectin, an anti-inflammatory adipokine, and decreased the mRNA levels of the inflammatory markers’ leptin, IL-6 and IL-17, in inflamed mesenteric adipose tissue from patients with Crohn’s disease [52]. Boccara et al. showed that HIV protease inhibitors (PI) increased AGT mRNA expression and AT1R protein levels as well as Ang II signalling through an ERK1/2-dependent pathway in SAT, and these effects were blocked by ARB (irbesartan and telmisartan) [53].

Adipocyte paracrine interactions with other cell types were also studied [54–56]. Blumensatt et al. investigated the effect of conditioned medium (CM) produced by human EAT from patients with type 2 diabetes on Lewis rat cardiomyocytes [54]. EAT secretory products increased inflammatory markers and impaired cardiomyocyte function by reducing sarcomere shortening and increased miR-208a expression. The effect was inhibited by the addition of ARB (losartan) to EAT-conditioned medium. Rasha et al. treated breast cancer cells with CM from human adipocytes pretreated with ACE inhibitors and eicosapentaenoic acid (EPA) [55]. The protective effect of ACE inhibitors in lessening adipocyte inflammation in breast cancer cells was increased with EPA. In a second study, Rasha et al. demonstrated that Ang II did not directly alter the secretion of inflammatory cytokines by breast cancer cells [56]. However, treatment of these cells with CM from Ang II-treated adipocytes did increase their secretion of pro-inflammatory proteins. CM from adipocytes treated with an ARB (telmisartan) and ACE inhibitor (captopril) significantly reduced this effect.

There has been interest in the role of ACE-2 in adipose tissue inflammation [57–59]. de Oliveira et al. reported irisin, an anti-inflammatory cytokine, reduced expression of genes that regulate ACE-2 cleavage in SAT [57]. Patel et al. found ACE-2 protein suppressed EAT inflammation [58]. Pinheiro et al. [59] noted increased expression of AGT and ACE-1 mRNA, along with that of TNF-α and IL-6, in VAT of obese and malnourished individuals [59]. No significant differences were found for ACE-2 expression between groups.

#### 3.2.4. RAS components associated with blood pressure

RAS components have been implicated in the control of blood pressure [60–66] (Table 4). Factors associated with increased blood pressure and AGT gene expression have been reported [60–64]. Serazin et al. treated SAT with cAMP analogue 8Br-cAMP and this increased ATG mRNA and protein levels [60]. Prat-Larquemin et al. demonstrated that AGT secretion by SAT adipocytes was not related to adipocyte size, BMI, blood pressure or M235T AGT gene polymorphism [61]. Sarzani et al. analysed AGT promotor variants in human kidney cortex, medulla and perirenal adipose tissue [62]. They found that AGT promotor variants influenced transcriptional activity in a tissue-specific manner and the presence of AGT promotor variants at −175 and −163 were most highly expressed in the perirenal adipose tissue depot. Park et al. analysed human AGT promoter polymorphisms for AGT mRNA expression and found a significantly higher expression of AGT mRNA derived from the −20C allele in comparison to the −20A allele in SAT but not omental VAT [63]. Gorzelniak et al. found AGT mRNA expression was significantly lower in adipocytes from obese subjects irrespective of blood pressure [64]. Renin, ACE-1 and AT1R mRNA expression were significantly higher in patients with obesity and hypertension. Hydrocortisone increased AT1R mRNA and protein expression in a time- and dose-dependent manner. Expression of these genes was not affected by insulin, thyroxine, oestradiol or Ang II.

Malinowski et al. reported that internal thoracic artery rings without, versus with perivascular adipose tissue, contracted more strongly in response to Ang II [65]. Perivascular adipose tissue decreased Ang II-stimulated contraction by releasing nitric oxide (NO) and prostacyclin-dependent anticontractile factor. Pleural adipose tissue did not influence internal thoracic artery reactivity in Ang II. Ehrhart-Bornstein et al. found that treatment of adrenocortical cells (NCI-H295R) with conditioned medium (CM) from isolated adipocytes significantly increased aldosterone secretion by adrenocortical cells [66]. This effect is independent of the adipose Ang II. The stimulatory effect of adipocyte CM in the presence of an ARB (valsartan) was unaltered.

## 4. Discussion

Our scoping review provides a preliminary assessment of *in vitro* studies describing the local expression and molecular actions of RAS in human adipose cells and tissue. After searching six databases, we retrieved only one *in vitro* human adipose article investigating ACE-2 [33]. Thus, there appeared to be a gap in the existing literature related to this important molecule, especially since ACE-2 has been identified as a cellular receptor for the SARS-CoV-2 virus [67]. In addition, obesity was recognized by the WHO as a risk factor for severe COVID-19 outcomes [68]. We postulated more articles would be published on human adipose ACE-2, after our initial search. Another PubMed search was performed, from inception to 30 July 2021, to determine if more articles had been published on human adipose ACE-2. Our PubMed search identified 14 new articles. All 14 articles were included in our scoping review to reflect a more current assessment of the literature on human adipose ACE-2.

In total, there were 50 articles included for this scoping review [17–66]. Articles published on human adipose RAS mainly reported on the expression of its components, as well as its impact on differentiation, inflammation, and its relationship to blood pressure regulation. Several publications have indicated that molecular mechanisms involving human adipose RAS were associated with conditions such as obesity, inflammation, hypertension, type 2 diabetes, cardiac disease, cancer, and COVID-19. Overall, the body of information we evaluated underscored the importance of adipose RAS in health and disease.

### 4.1. Expression of RAS components

Our scoping review confirmed that all the components of systemic RAS were expressed locally by preadipocytes and adipocytes in human adipose tissue at the mRNA and protein level. A variety of adipose tissue depots were investigated, with SAT being the most frequent. Studies comparing VAT and SAT found a higher expression of RAS components in VAT [23,28,30] There were 17 studies which used all female donors and 3 studies which used all male adipose tissue donors (Tables 1–4). Studies which compared male and female ACE-2 mRNA expression found no differences [17,19,22,24]. In comparison to preadipocytes, adipocytes expressed higher mRNA levels of AGT, renin, AT1R and ACE-2 as well as higher protein levels of Ang II, Ang (1–7) and MasR [29,31,33,40]. On the other hand, preadipocytes expressed higher levels of ACE-1 and (pro)renin receptors in comparison to adipocytes [28–30,40]. Studies have shown that the stage of differentiation influenced the extent of expression differently depending on the component of interest. This might reflect different functions of these molecules in the progenitor versus the mature cell.

Several studies examined human adipose RAS in the setting of obesity and changes in BMI. Changes in RAS parameters might correlate with the metabolic state associated with the lean versus obese phenotype. Engeli et al. found that there were higher circulating levels of AGT, renin, aldosterone, and ACE-1 in obese versus lean human participants [69]. Weight reduction of 5% body weight in obese subjects lowered these levels. The adipocyte expression of mRNA of renin, ACE-1, and AT1R was higher in adipocytes derived from individuals with obesity [28,59,64]. In the case of AGT mRNA expression, there was wide inter-individual variability [59,61,64].

ACE-2 expression in adipose tissue is altered by obesity, and obesity is considered a risk factor for COVID-19 [68,70]. Our search parameters identified only one article that directly studied the adipose of ACE2 expression as a function of COVID-19 infection. Favre et al. observed that VAT, but not SAT, expression of ACE-2 mRNA was associated with severity of COVID-19 symptoms [23]. During the revision process of our review, a very recent article reported actual SARS-CoV-2 infection of human adipose tissue; in this study, it appeared that ACE-2 was not the main mediator of viral uptake [10].

Other articles, with the advent of the COVID-19 pandemic, were motivated to study ACE2 given its previously described role as a viral receptor for SARS-CoV-2. SAT or EAT ACE-2 mRNA expression was higher in individuals with elevated BMI [21,22,24,58]. Individuals with cardiovascular disease and obesity with type 2 diabetes were found to express higher levels of adipose ACE-2 mRNA in EAT than those without diabetes [24,26] Weight reduction decreased adipose ACE-2 mRNA expression [21,22]. RNAseq transcriptome database analysis found that ACE-2 mRNA levels decreased for individuals post-RYGB surgery. It was suggested RYGB surgery could benefit individuals with obesity by reducing the risk for severe SARS-CoV-2 infections [21].

In a cross-sectional analysis, Pinheiro et al. [59] found only a slight increase in ACE-2 expression at higher BMI that was not significant.

Another area of interest early on in the COVID-19 pandemic was the risk of COVID-19 for individuals using ARBs [71]. Studies in rats had indicated that the use of ARBs had the potential to upregulate ACE-2 mRNA expression [72,73]. Articles in our review approached this issue by examining ACE-2 expression in the context of ARB treatment. One study indicated that patients with cardiovascular disease and diabetes who were treated with ARBs had higher ACE-2 mRNA expression in EAT but not in SAT [24]. Another study investigating SAT from patients with or without diabetes showed treatment with an ARB (valsartan had no correlation with ACE-2 mRNA expression in SAT) [26]. More studies on adipose depot-dependent modifications and molecular mechanisms related to regulation of ACE-2 mRNA levels by ARBs are needed. Although higher levels of ACE-2 may provide more cellular receptors for SARS-CoV-2, higher levels of ACE-2 may also be theoretically protective by counterbalancing the negative effects of ang II.

### 4.2. Differentiation and RAS components

Adipogenesis is an important process for maintaining proper adipose tissue function in healthy individuals [6,74]. Local adipose RAS has been shown to play a critical role in the regulation of adipogenesis [32]. Obesity-related complications such as type 2 diabetes have been associated with adipocyte dysfunction due to adipocyte hypertrophy. The inability of adipose tissue to expand through adipocyte hyperplasia can cause adipocyte hypertrophy. This can in turn lead to adipose dysfunction and loss of insulin sensitivity [6,74]. The majority of the studies we identified concluded that Ang II inhibited adipogenesis [31–41]. The anti-adipogenic effect of Ang II was mediated through its interaction with AT1R. *In vitro* studies have indicated that the adipogenesis is stimulated through the PPAR-γ pathway and inhibited through the ERK1/2 pathway [38]. Stimulation of the ERK1/2 pathway by Ang II has also been shown to inhibit insulin stimulated Akt phosphorylation [37]. Ang II/AT1R decreased the activity of PPAR-γ and increased the activity of ERK1/2 [38]. The addition of ARBs blocked the anti-adipogenic effects of Ang II by activation of PPAR-γ [39]. The anti-adipogenic effects of Ang II appeared to be significantly greater in VAT-derived adipocytes from individuals with higher BMIs [35,37]. Adipocyte progenitor cells isolated from individuals with higher BMIs displayed less of an adipogenic response [35,37]. Furthermore, several studies indicated that the anti-adipogenic effects of Ang II were associated with insulin resistance [36–39,41]. The only article that reported Ang II promoted adipogenesis was by Sarzani et al. [42]. They suggest differences in AT1R versus AT2R, as well as concentrations of reagents, may account for their findings.

The expression of AGT gene expression increased during adipogenesis. The presence of AT1R and AT2R was also required for adipogenesis [32,40]. Both preadipocytes and adipocytes were found to express AT1R proteins [31,32,41]. Mature adipocytes expressed AT2R, however, not all ADSCs expressed AT2R [32]. The adipogenic potential of ADSCs corresponded to AT2R expression [32]. Gene expression of AT1R and AT2R was dependent on the stage of adipogenesis increasing initially and then decreasing in the later stages [40]. The Ang (1–7)/MasR pathway has also been found to be involved in the autocrine regulation of adipogenesis. The inhibitory effects of Ang II/ATR1 could be offset by Ang (1–7)/MasR [41]. Our scoping study showed a gap in knowledge for the mechanistic role of human adipose AT2R and Ang(1–7)/MasR in adipogenesis.

Adipose tissue depots are heterogeneous. SAT has been shown to contain white adipocytes as well as clusters of beige adipocytes [75]. Beige adipocytes share the same thermogenic capacity as brown adipocytes, which is important for heat production and energy expenditure. Individuals with obesity have been found to have lower amounts of beige adipocytes [76,77]. Beige adipocytes are emerging as novel therapeutic targets for the treatment of obesity-related diseases [33]. Than et al. [33] enhanced brown adipogenesis by promoting AT2R signalling through inhibition of AT1R inhibition. Thyroid hormone T3 promoted brown adipogenesis by selectively stimulating AT2R without altering the expression of AT1R, ACE-1, ACE-2 or AGT [33]. Further research is required to investigate the formation of beige adipocytes and the role of AT2R in this process.

ADSCs are a promising stem cell type for cell-based therapies. ADSCs are an abundant and accessible source of adult stem cells with the ability to differentiate along multiple lineage pathways [78]. Animal studies have indicated that the injection of ADSCs improves cardiac function through differentiation into cardiomyocytes and vascular cells through paracrine pathways [79,80]. In our scoping review, we found two studies, which used precursor stem cells for *in vitro* trans-differentiation of cardiomyocytes with limited success [43,44]. There will likely be further advancements in this area in the future.

### 4.3. RAS and adipose inflammation

With prolonged positive energy balance, hypertrophied adipocytes reach a threshold that causes cellular stress and initiates an inflammatory programme. Inflamed adipocytes secrete pro-inflammatory cytokines, which can disrupt the normal function of adipose tissue as well as that of remote organs [81]. *In vitro* studies showed that adipocyte pro-inflammatory cytokines had the ability to influence the growth and migration of breast cancer cells [55,56]. Chronic low-grade inflammation in adipose tissue has been shown to be a risk factor for the development of insulin resistance and type 2 diabetes in individuals with obesity [82]. Many molecular mechanisms operating within adipocytes have been suggested as possible regulators of inflammation, including ER stress, hypoxia and cellular senescence.

Adipose RAS has been shown to play a role in adipose tissue inflammation. Local administration of Ang II induced tissue hypoxia and increased the expression of inflammatory markers [45]. Both insulin and TNF-α increased the secretion of AGT and Ang II secretion by adipocytes [46]. In preadipocytes, exposure to Ang II increased mitochondrial ROS and increased markers associated with senescence [49]. In adipocytes, Ang II exposure was shown to increase ER stress and increase levels of NF-κB and its downstream target IL-6 [47]. Ang II could also stimulate the release of pro-thrombotic plasminogen activator inhibitor-1 (PAI-1) [50]. The undesirable effects of Ang II were inhibited by treatment with ARBs, which indicated that the pro-inflammatory pathways were associated with Ang II interaction with AT1R [47–51]. Additionally, one study found that treatment with ARB reduced inflammatory markers secreted by mesenteric adipose tissue from patients with Crohn’s disease [52].

Conversely, ACE-2 has been associated with an anti-inflammatory role. Patients with higher levels of ACE-2 in epicardial tissue had a lower risk of inflammation-related issues [58]. Another study associated the reduction in ACE-2 cleavage in SAT with the anti-inflammatory effects of irisin [57]. Furthermore, ACE-2 enzyme can convert Ang II to Ang(1–7) [41]. The Ang(1–7)/MasR pathway has been associated with the counter-regulation of AngII/AT1R [41]. Thus, an increase in conversion of Ang II to Ang (1–7) could have the potential to reduce inflammation.

### 4.4. RAS components associated with blood pressure

Systemic RAS is important for blood pressure control. Various components of human adipose RAS have been studied for their possible role in systemic blood pressure regulation. AGT is a precursor of Ang II, the principal effector hormone for blood pressure regulation. A number of studies have investigated the role of adipose AGT for blood pressure regulation in humans. Sympathetic stimulation of adipocytes from SAT with a cAMP analogue was shown to increase AGT gene expression [60]. However, there were many variables associated with AGT gene expression, which included a wide inter-individual as well as tissue-specific variability [59,61,62,64,64]. Harte et al. found Ang II that was produced locally in abdominal SAT and was a significant source of Ang II in the systemic circulation [46] One study found that AGT secretion by SAT human adipocytes was not associated with increased blood pressure [61]. Additionally, Ang II reduced thoracic ring contraction through the release of NO in perivascular adipose tissue [34,65]. Another study found that hypertension was associated with lower levels of AGT and higher levels of renin, ACE-1 and AT1R mRNA expression in SAT [64]; the inconsistent findings among these studies have been attributed to differences in study population

### 4.5. Strengths and limitations of the study

The review applied a systematic and rigorous search strategy to retrieve relevant articles according to the research objectives. We used a scoping review to identify the nature and breadth of the current evidence available. An assessment of the quality of the included studies is not the usual expectation of scoping reviews and thus was not included, which is a potential limitation of our study. However, we selected only peer-reviewed primary literature as part of our screening criteria. Our study summarizes scientific findings and highlights significant heterogeneity in several areas including adipose tissue source and variations in the methodology used to characterize adipose RAS. It identifies literature gaps and suggests some directions for future research initiatives on human adipose RAS.

A major limitation of our study was that our database search was conducted on 24 June 2020. Our study showed the state of research available at an early stage of the pandemic. Thus, it would be informative in a future study to compare our study with the state of research currently available as the pandemic has progressed. Our study was also limited to articles published in the English language. Owing to the broad scope of study methodologies used to analyse adipose tissue, the database search strategy was limited by including only studies with the term ‘*in vitro’* in the MESH heading used in the search strategy. Furthermore, the COVID-19 pandemic has generated a new interest in adipose ACE-2, and there has been an escalation in publications. Although this scoping review does not capture all articles due to this recent rapid acceleration, it does serve as a landmark of current information, and has the potential to become a reference point for future investigations in this area.

## 5. Conclusions

This scoping review was conducted as a preliminary assessment of the state of the literature published related to *in vitro* human adipose RAS from 2001 to 2021. Adipose RAS plays an important role in adipose tissue homoeostasis, differentiation, obesity, inflammation, and hypertension. Research on human adipose RAS has been rapidly evolving since the onset of the pandemic. There has been increased utilization of bioinformatics and RNAseq transcriptome databases to study ACE-2, the cellular receptor for the SARS-CoV-2 virus.

We gathered relevant scientific evidence on the role of RAS in various cellular processes in human adipose tissue/adipocytes, primarily associated with expression of local RAS components, adipocyte differentiation and trans-differentiation, inflammation and molecular mechanisms involved with blood pressure control. Obesity-related changes in adipose RAS were frequently studied. The undesirable effects of Ang II/AT1R which included the inhibition of adipogenesis, and inflammation could be effectively blocked through the usage of ARBs. More studies are required to determine the effect of ARBs on ACE-2. Stimulation of beige adipocytes for the treatment of obesity appeared promising; however, there was a gap in knowledge of the molecular mechanism for AT2R involvement. We also identified a paucity of papers addressing the human adipose Ang (1–7)/MasR pathway, a counterbalance to the Ang II/AT1R pathway. Investigating the potential role of different components of human adipose RAS, the signalling pathways that are associated with its expression, as well as identifying adipocyte interactions with cardiomyocytes, cancer cells, or other cell types, will open new avenues for research and allow us to better understand the role of human adipose RAS in health and disease.

## Supplementary Material

Supplemental Material
