# Supplementary material for: In vitro studies of the renin-angiotensin system in human adipose tissue/adipocytes and possible relationship to SARS-CoV-2: a scoping review
Source: Adipocyte. 2023 Mar 27;12(1):2194034. doi: 10.1080/21623945.2023.2194034 (PMC10054178; doi:10.1080/21623945.2023.2194034)
Supplement: Supplemental Material [file KADI_A_2194034_SM7464.docx]

**Abbreviations**

5-AZA

5-azacytidine

ACE-1

Angiotensin converting enzyme 1

ACE-2

Angiotensin converting enzyme 2

ACE inhibitor

Angiotensin converting enzyme inhibitor

ad-MSCs

Adipose-derived mesenchymal stem cells

ADSC

Adipose-derived stem cells

Ang (1-7)/MasR

Angiotensin (1-7)

Ang I

Angiotensin I

Ang II

Angiotensin II

AGT

Angiotensinogen

ARBs

Angiotensin receptor blockers

AT1R

Angiotensin type 1 receptors

AT2R

Angiotensin type 2 receptors

BC

Breast cancer

BMI

Body mass index

cAMP

Cyclic adenosine monophosphate

CM

Conditioned medium

EAT

Epicardial adipose tissue

EPA

eicosapentaenoic acid

IL-6

Interleukin-6

IL-17

Interleukin-17

MasR

Mas receptor

MiR-208a

MicroRNA 208a

MSC

Mesenchymal stem cells

NF-𝜅B

Nuclear factor kappa B

OAT

Omental adipose tissue

NO

Nitric oxide

PPAR-𝛾

Peroxisome proliferator-activated receptor gamma

PAI-1

Plasminogen activator inhibitor 1

PRISMA-ScR

Preferred Reporting Items for Systematic Reviews and Meta-Analyses -- Extension for Scoping Reviews

RAS

Renin-angiotensin system

ROS

Reactive oxygen species

RYGB

Roux-en-Y gastric bypass

SAT

Subcutaneous adipose tissue

SA-𝛽-gal

Senescence-associated beta-galactosidase

TNF-𝛼

Tumour necrosis factor alpha

VAT

Visceral adipose tissue
